# Developmental synapse pathology triggered by maternal exposure to the herbicide glufosinate ammonium

**DOI:** 10.3389/fnmol.2023.1298238

**Published:** 2023-11-30

**Authors:** Hironori Izumi, Maina Demura, Ayako Imai, Ryohei Ogawa, Mamoru Fukuchi, Taisaku Okubo, Toshihide Tabata, Hisashi Mori, Tomoyuki Yoshida

**Affiliations:** ^1^Department of Molecular Neuroscience, Faculty of Medicine, University of Toyama, Toyama, Japan; ^2^Research Center for Idling Brain Science, University of Toyama, Toyama, Japan; ^3^Department of Radiology, Faculty of Medicine, University of Toyama, Toyama, Japan; ^4^Laboratory of Molecular Neuroscience, Faculty of Pharmacy, Takasaki University of Health and Welfare, Gunma, Japan; ^5^Laboratory for Biological Information Processing, Faculty of Engineering, University of Toyama, Toyama, Japan; ^6^Research Center for Pre-Disease Science, University of Toyama, Toyama, Japan

**Keywords:** developmental neurotoxicity, glufosinate ammonium, maternal pesticide exposure, synapse formation, synaptic organizers

## Abstract

Environmental and genetic factors influence synapse formation. Numerous animal experiments have revealed that pesticides, including herbicides, can disturb normal intracellular signals, gene expression, and individual animal behaviors. However, the mechanism underlying the adverse outcomes of pesticide exposure remains elusive. Herein, we investigated the effect of maternal exposure to the herbicide glufosinate ammonium (GLA) on offspring neuronal synapse formation *in vitro*. Cultured cerebral cortical neurons prepared from mouse embryos with maternal GLA exposure demonstrated impaired synapse formation induced by synaptic organizer neuroligin 1 (NLGN1)–coated beads. Conversely, the direct administration of GLA to the neuronal cultures exhibited negligible effect on the NLGN1-induced synapse formation. The comparison of the transcriptomes of cultured neurons from embryos treated with maternal GLA or vehicle and a subsequent bioinformatics analysis of differentially expressed genes (DEGs) identified “nervous system development,” including “synapse,” as the top-ranking process for downregulated DEGs in the GLA group. In addition, we detected lower densities of parvalbumin (Pvalb)-positive neurons at the postnatal developmental stage in the medial prefrontal cortex (mPFC) of offspring born to GLA–exposed dams. These results suggest that maternal GLA exposure induces synapse pathology, with alterations in the expression of genes that regulate synaptic development via an indirect pathway distinct from the effect of direct GLA action on neurons.

## 1 Introduction

Pesticides, as agricultural chemicals, are used to protect crops from diseases, pests and weeds, and to increase agricultural productivity. To maximize the benefits of pesticides and minimize the disadvantages, it is necessary to evaluate the risk of impact of pesticides on humans (U.S. Environmental Protection Agency, 2023). There have been concerns that exposure to pesticides during the perinatal period affects the early stages of neurodevelopment, contributing to adverse outcomes (Roberts et al., 2019; von Ehrenstein et al., 2019), thereby highlighting a possibility that the transfer of pesticides to the fetal brain via maternal exposure is an environmental factor that could result in developmental disorders. However, the molecular pathogenic mechanisms triggered during the neurodevelopment due to exposure to relatively low-concentration pesticides remain unknown owing to a higher degree of symptom concealment compared with that in accidental cases with acute toxicity. Based on numerous studies with experimental animals that assessed the developmental neurotoxicity caused by pesticide exposure, data have accumulated regarding primary changes within neuronal cells, including alterations in intracellular signaling, metabolism, and gene expression as well as eventual adverse outcomes, such as learning and behavioral abnormalities in individual animals. For example, perinatal exposure to pesticide induced sexually dimorphic social behavioral alteration with modulation of neuroendocrine gene expression (Venerosi et al., 2015), alteration of dopaminergic system, attention deficit hyperactivity disorder-like behavior (hyperactivity, working memory and attention deficit, and impulsive-like behavior) (Richardson et al., 2015), alteration of neurogenesis, neuronal activity, and increased anxiety level and locomotor activity (Maeda et al., 2021). However, secondary or tertiary pathomechanisms linking primary cellular-level changes to the final phenotypes of individual animals remain to be clarified. In particular, there is a need to evaluate the effect of pesticide exposure on neuronal synapses, which form the basis of neural circuits and can change plastically in response to various external stimuli (Vester and Caudle, 2016).

Glufosinate ammonium (GLA) is a key herbicide that is being increasingly used to manage glyphosate-resistant weeds (Takano and Dayan, 2020; Donthi and Kumar, 2022). Its action mechanism in plants involves inhibiting glutamine synthetase (GS), which consequently leads to reactive oxygen species accumulation and lipid peroxidation, in turn resulting in rapid cell death (Takano and Dayan, 2020). However, GLA is structurally similar to the neurotransmitter glutamate; thus, there is a growing concern about its effect on the central nervous system (CNS) in mammals, including humans. The effects by acute exposure include memory impairment and cognitive dysfunction in human (Park et al., 2020; Lee and Kang, 2021). Maternally exposed GLA is reported to cause reduced locomotor activity, impaired memory formation, and an autism-like behavior in offspring in mice (Laugeray et al., 2014; Dong et al., 2020; Oummadi et al., 2023). Previously, we established a novel transgenic (Tg) mouse strain termed *Arc-Luc* Tg to visualize neuronal activity-dependent *Arc* expression *in vivo* (Izumi et al., 2017). Arc is one of the molecules responsible for synaptic regulation among neurons, and its expression is induced in a neuronal activity-dependent manner (Okuno et al., 2018). By tracking bioluminescence signals in this Tg mouse, we detected a decrease in Arc-Luc expression in the brain following subchronic exposure to GLA during the postnatal developmental stage (Izumi et al., 2019). This result suggests that GLA exposure during the neurodevelopmental stages can modulate the formation of neural circuits and synaptic functions, ultimately resulting in learning and behavioral disorders at the individual level.

The mammalian brain comprises billions of neurons that form networks through synapses with each other (Herculano-Houzel, 2009). The formation of new synapses takes several hours, and up to 10% of synapses are remodeled daily (Changeux and Danchin, 1976; Lichtman and Colman, 2000; Yang et al., 2009). Numerous studies have revealed that synapse formation does not occur randomly between adjacent neurons; rather, it is precisely regulated via cell-adhesion molecules known as synaptic organizers, such as neurexins (NRXNs) and neuroligins (NLGNs) (Südhof, 2017). Synaptic organizers have unique functions such as inducing presynaptic and postsynaptic differentiation and regulating synaptic target selection. We established an artificial synapse-formation assay system to quantitatively analyze the synapse-inducing activity of cultured neurons under various conditions using synaptic organizer–coated beads (Yoshida et al., 2011, 2012, 2021; Yamagata et al., 2015; Goto-Ito et al., 2018).

Herein, we applied the synapse-formation assay to cultured mouse cerebral cortical neurons to evaluate the effect of GLA exposure on synapse formation. The application of NLGN1–coated beads to cultured cortical neurons obtained from embryos with maternal GLA exposure decreased the accumulation of a presynaptic marker, Bassoon, around the beads compared with that observed in cultured neurons obtained from maternally saline vehicle–treated embryos. In addition, we also confirmed that this change was not detected in cultured neurons with subchronic direct exposure to GLA. Consistently, we identified differentially expressed genes (DEGs) extracted from a transcriptome analysis of neurons with maternal GLA exposure related to the biological processes of nervous system development and synapse components. Furthermore, we found that offspring that were maternally exposed to GLA exhibited a lower density of parvalbumin (Pvalb)-immunoreactive interneurons during the postnatal developmental stage. Thus, our study showed that maternal GLA exposure induces a developmental synapse pathology through pathways that differ from those underlying the direct action of GLA on neurons.

## 2 Materials and methods

### 2.1 Animals and GLA administration

**FIGURE 1:**
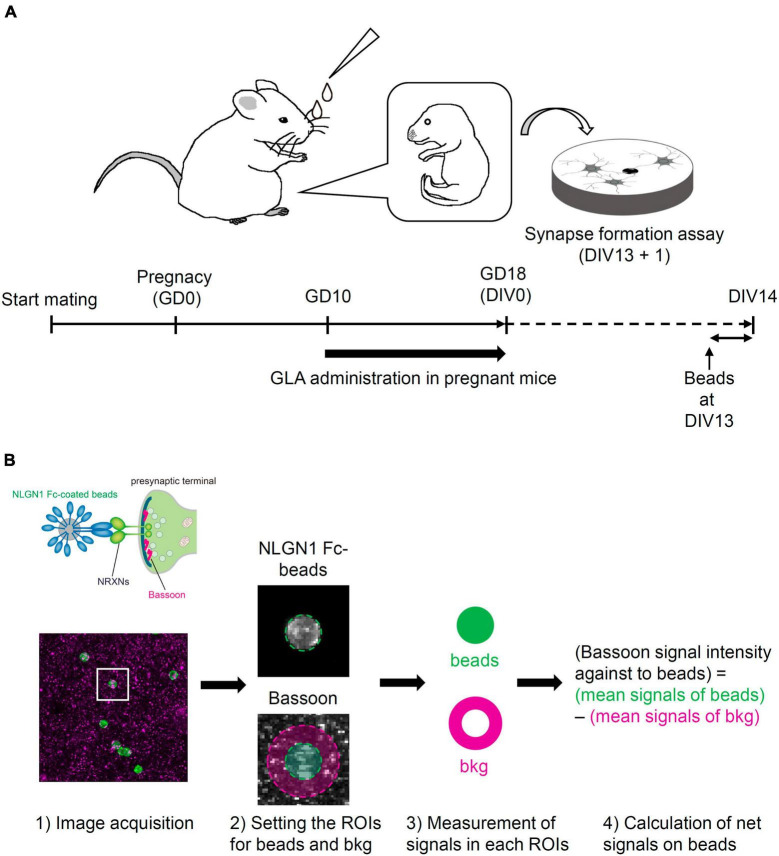
Schematic illustration of the experimental procedure and the analysis of synapse-formation assay. **(A)** Experimental procedure of synapse-formation assay after maternal GLA exposure. Pregnant mice were treated by intranasal administration of GLA (0.8 mg/kg) from GD10 to GD18. Cultured cortical neurons prepared from GD18 embryos were subjected to synapse-formation assay at DIV13. Animals in the control group were treated with same volume of saline-vehicle instead of GLA. **(B)** Overview of the analytical steps for synapse-formation assay, (1) schematic of presynaptic differentiation by NLGN1 Fc–coated beads (upper), acquisition of images of NLGN1 Fc–coated beads (green) and Bassoon signals (magenta) (lower), (2) setting the region of interest (ROI) for each bead and background (bkg), example images are from the inset in (1), (3) measurement of Bassoon signal intensity in each ROI, (4) calculation of net Bassoon signal intensity on the NLGN1 Fc–coated beads given by subtracting mean Bassoon signal intensity on the bkg area from that on the bead area. Detailed procedure is also described in section “Materials and methods.”

C57BL/6N mice (CLEA Japan, Inc.) at 3 weeks of age (3W) and ICR mice (Japan SLC, Inc.) at 10W were purchased and used in this study. To match the fetal development between the control and GLA groups, frozen two-cell embryos from C57BL/6N mice were prepared via *in vitro* fertilization and transplanted in pseudo-pregnant ICR mice according to a method reported previously (Miyao et al., 2020). After recovery from the operation, the transplanted mice were housed individually. Mice in the GLA group were intranasally administered 10 μL GLA (Glufosinate Ammonium Standard; FUJIFILM Wako Pure Chemical Corporation) dissolved in saline using a pipette [0.8 mg/kg of body weight (BW), 5 μL/nostril] according to a previously reported method (Laugeray et al., 2014). The animals in the negative control group received an equal volume of a vehicle. The treatment was performed (1) from gestation day (GD) 10 to GD18 (GD0 being the day on which the two-cell embryos were transplanted in the recipients) for the primary culture of cortical neurons obtained from embryos at GD18 (Figure 1A); and (2) from GD10 to postnatal day (PND) 14 (PND0 being the day on which the pups were delivered or rescued via cesarean section and fostering) for analyzing the brain of the offspring (Figure 5A).

The animal care and experimental protocols were approved by the Ethics Committee for Animal Experiment of the University of Toyama (Approval No. A2023MED-05). All personnel members engaged in animal care and experimentation were appropriately trained according to the Guidelines for the Care and Use of Laboratory Animals of the University of Toyama. The mice were maintained under standard conditions (12/12 h light/dark cycle) at the Animal Resource Research Center, with free access to water and diet. Embryo transfer enabled us to match the fetal development between the control and GLA groups without keeping several pairs under continuous mating, thereby reducing the number of mice used, animal maintenance space, and time and cost for animal experiments. Anesthesia via isoflurane inhalation (1.5% in oxygen) was administered to pseudo-pregnant ICR mice for surgical embryo transfer. Euthanasia with cervical dislocation was performed for *in vitro* fertilization and cesarean section.

### 2.2 Synapse-formation assay

Primary cortical neurons were prepared from maternal GLA–exposed or vehicle control–treated embryos at GD18 as described previously (Yoshida et al., 2011). Pregnant mice were euthanized via cervical dislocation and the embryos were collected. Multiple cultures were prepared from four to five embryos from each pregnant mouse for the synapse-formation assay. Dispersed cells were plated at a density of 5 × 10^5^ cells/well on cover slips in 24 well plates and maintained in a Neurobasal medium (Thermo Fisher Scientific Inc.) with B-27 supplementation (Thermo Fisher Scientific Inc.) at 37°C with 5% CO_2_. One-fourth of the culture medium was replaced with fresh medium every 3 days. For the synapse-formation assay, human Fc or mouse NLGN1 proteins fused to human Fc were immobilized on protein A–conjugated magnetic beads (smooth surface, 4.0–4.5 μm in diameter; Spherotech, Inc.). These magnetic beads were added to the cultured neurons at day *in vitro* (DIV) 13. After 24 h, the bead–neuron co-cultures were fixed for fluorescent immunostaining. For the quantification of immunostaining signals for the presynaptic marker Bassoon in the co-cultures, the fluorescent mean densities within a circle with a diameter of 7 μm enclosing the NLGN1–coated beads were measured. Subsequently, the optical mean densities of the surrounding regions within a circle with a diameter of 14 μm were subtracted as the background signals of the beads to obtain the net fluorescent signals on the beads. The steps of quantitative analysis for the synapse-formation assay are diagrammed in Figure 1B. When the net fluorescent signal on beads was <0, the signal was regarded as 0 and used for statistical analysis. We confirmed that the background signals for Bassoon were comparable across the experiments to monitor the culture conditions (Supplementary Figure 2C).

### 2.3 Immunofluorescent staining

For immunocytochemistry using the cultured neurons, the neurons on the cover slips were fixed with 4% paraformaldehyde (PFA) and 4% sucrose in phosphate-buffered saline (PBS) for 20 min at room temperature (RT): 15–25°C. The neurons were washed in PBS and permeabilized with 0.25% Triton X-100 in PBS for 5 min. After washing, the neurons were incubated in 10% donkey serum in PBS for 1 h at RT. Subsequently, the cover slips were incubated with primary antibodies overnight at RT followed by incubation with fluorescent secondary antibodies for 2 h at RT.

For immunohistochemistry using brain sections, offspring mice at PND14 and PND21 were deeply anesthetized via isoflurane inhalation (5% in oxygen) and transcardially perfused with ice-cold PBS followed by 4% PFA in 0.1 M phosphate buffer (PB, pH 7.4). Serial coronal sections (50-μm thickness, bregma from 2.09 mm to 1.77 mm) were prepared using a vibratome (Leica VT1000S, Leica Microsystems) based on the “The mouse brain in stereotaxic coordinates” (Paxinos and Franklin, 2013). The sections were preincubated with a blocking buffer (0.5% Triton X-100 and 1% BSA in PBS) for 1 h at RT. Next, the sections were incubated with primary antibodies diluted in the blocking buffer overnight at 4°C and then washed in PBS and incubated with fluorescent secondary antibodies diluted in the blocking buffer for 1 h at RT. The number of NeuN- and Pvalb-immunoreactive cells in the medial prefrontal cortex (mPFC) were counted. The percentage of Pvalb-immunoreactive cells was calculated as the number of double-labeled cells/total NeuN-immunoreactive cells.

The following antibodies were used here: goat anti-Pvalb (1:1000, Frontier Institute, MSFR105240), mouse anti-NeuN (1:300, Millipore, MAB377), mouse anti-Bassoon (1:300, Stressgen, ADI-VAM-PS003F), rabbit anti-human Fc (1:1000, Rockland, 609-4103), Alexa fluor 647–conjugated donkey anti-goat (1:300, Invitrogen, A-21447), Alexa fluor 488–conjugated donkey anti-rabbit IgG (1:300, Invitrogen, A-21206), and Alexa fluor 488–conjugated donkey anti-mouse IgG (1:300, Invitrogen, A-21202) antibodies. All images were acquired using a confocal laser-scanning microscope (Leica TCS-SP5II, Leica Microsystems). Three-dimensional stacks were acquired using a step size of 1.0 μm for cultured neurons and 2.0 μm for brain sections.

### 2.4 Microarray analysis

Cultured neurons at DIV6, DIV10, and DIV14 without the addition of NLGN1–coated beads were used for microarray analysis. Total RNA was extracted from cells using the TRIzol reagent (Thermo Fisher Scientific Inc.) followed by treatment with DNase I (Qiagen K.K.) and further purification using an RNeasy total RNA Extraction kit (Qiagen K.K.). The concentration and quality of the RNA were measured using NanoDrop 2000 (Thermo Fisher Scientific Inc.) and Bioanalyzer 2100 with RNA 6000 nano kit (Agilent Technologies, Ltd.), respectively. The transcriptome analysis was performed using a GeneChip system with Clariom S Assay, mouse (Thermo Fisher Scientific Inc.) according to the manufacturer’s instructions. Shortly, 100 ng total RNA was used to synthesize ss-cDNA using a GeneChip WT Plus Reagent Kit (Thermo Fisher Scientific Inc.). Following fragmentation, biotin-labeled ss-cDNA was hybridized to the array at 45°C for 16 h. The arrays were washed, stained with a GeneChip Hybridization Wash and Stain Kit (Thermo Fisher Scientific Inc.), and scanned using GeneChip Scanner (Thermo Fisher Scientific Inc.). The raw intensity data were analyzed using Transcriptome Analysis Console software (Thermo Fisher Scientific Inc.) to extract the significant genes. The genes that passed the filter criteria of fold change of >1.3 (in linear space) with the log base 2 of signal intensity of >10 in control group, without genes in sex chromosomes, and a *P*-value of <0.05 (one-way between-subject ANOVA) were considered DEGs. For gene ontology assessment, a functional enrichment analysis of the DEGs was performed using the g:Profiler web server (Reimand et al., 2007). g:SCS threshold was selected as the significance threshold and was set to *P*-value of < 0.05.

### 2.5 Reverse transcription-quantitative PCR (RT-qPCR) analysis

RNA samples were reverse transcribed to cDNA using SuperScript IV First-Strand Synthesis System (Thermo Fisher Scientific Inc.) according to the manufacturer’s instructions. Gene expression was quantified using GeneAce SYBR qPCR Mix (Nippon Gene Co., Ltd.) on AriaMX real-time PCR system (Agilent Technologies, Ltd.). The following primers were used: 5′-ATTGT GTCCAGAAGGGCTCC-3′ and 5′-GCCAGCTTGTAGATGCGT TA-3′ for bassoon (*Bsn*); 5′-TCTCTGCAGGGCTAAGCAAC-3′ and 5′-TATAGATGTGCACGGCGACC-3′ for calcium channel, voltage-dependent, gamma subunit 3 (*Cacng3*); 5′-ACCAAGAAG TACGCCAAGGG-3′ and 5′-GCCTTGGAGGCTACGTCAAT-3′ for wolframin ER transmembrane glycoprotein (*Wfs1*); 5′-TCC AAGACGCCATGAAGCAT-3′ and 5′-CCCAGGTGTCACATTGT CCA-3′ for C–X3–C motif chemokine ligand 1 (*Cx3cl1*); and 5′-ACAGTCCATGCCATCACTGC-3′ and 5′-TAGGAACACGGA AGGCCATG-3″ for glyceraldehyde-3-phosphate dehydrogenase (*Gapdh*). The Cq value obtained for *Gapdh* was used to calculate the relative quantities of the target genes. All experiments were performed in duplicate and the specificity of the PCR products was confirmed using melting curves and gel electrophoresis.

### 2.6 Statistical analysis

Data are presented as mean ± the standard error of the mean (SEM) or mean ± the standard deviation (SD). Student’s *t*-test or one-way analysis of variance was used to determine the significance of the quantitative data for synapse-formation assay, immunostaining study, and RT-qPCR analysis. Statistical significance was set at a *P*-value of < 0.05. Detailed information is provided in each figure legend.

## 3 Results

### 3.1 Maternal GLA exposure disturbs presynaptic differentiation in cultured cortical neurons

Previously, we reported that Arc expression is modulated by postnatal GLA exposure in an age- and dose-dependent manner (Izumi et al., 2019). To identify the underlying mechanism of the effect of GLA exposure on neurodevelopment, we examined whether GLA exposure affects the formation of neuronal synapses.

**FIGURE 2:**
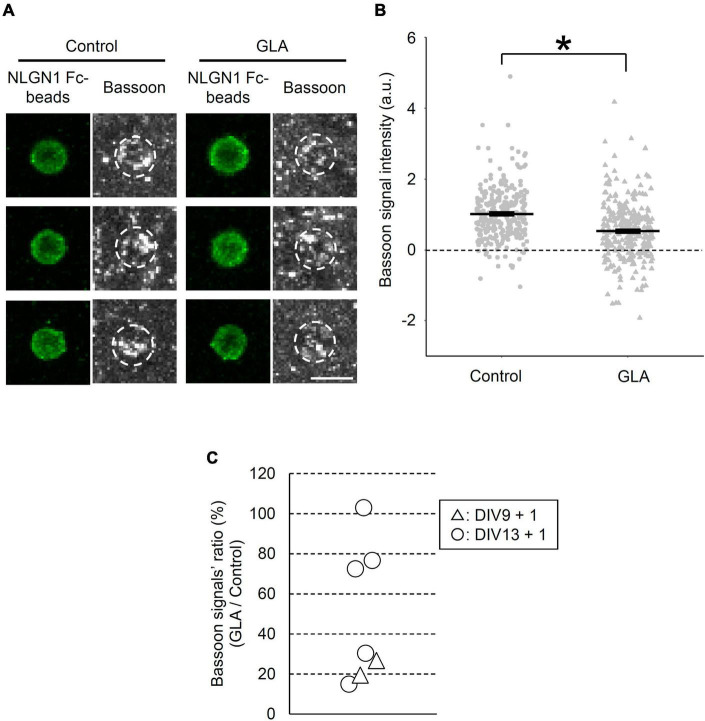
Effect of maternal GLA exposure on presynaptic differentiation. **(A)** Bead–neuron co-cultures of Control and GLA groups were immunostained for Bassoon (gray) and NLGN1 Fc–coated beads (green). White dashed circles outline beads. *Scale bar*, 5 μm. **(B)** Bassoon signal intensity on the surface of NLGN1 Fc–coated beads (*n* = 278 and 310 beads analyzed/3 cultures per litter/5 independent experiments for Control and GLA groups, respectively). Data are presented as dot plot of Bassoon signal intensity on NLGN1 Fc–coated beads in each experimental condition with mean ± SEM. *P < 0.05; two-tailed Student’s *t-*test. **(C)** Interlitter variations of the suppressive effect of maternal GLA exposure on presynaptic differentiation. Cultured cortical neurons from the GLA group display a decreasing trend of presynaptic differentiation by NLGN1 Fc–coated beads. Bassoon signal intensity of the GLA group was normalized by that of the control group in each experiment and shown as GLA/Control ratio (%). Each plot represents data of individual pregnant mice [DIV9 + 1 (*n* = 2 litters, triangle), DIV13 + 1 (*n* = 5 litters, circle)]. Six out of seven plots showed statistical significance (*P* < 0.05).

We employed an artificial synapse-formation assay wherein cultured cortical neurons from GD18 embryos that underwent maternal GLA exposure were incubated with beads coated with the recombinant extracellular domain of NLGN1 at DIV13 for 24 h (Figure 1A). In total, five experiments were conducted using independent sets of pregnant mice. Bead–neuron co-culture images acquired from the control and GLA groups were analyzed according to the steps shown in Figure 1B. NLGN1 is a well-known postsynaptic organizer that induces presynaptic differentiation through NRXNs after they are presented to cultured neurons (Scheiffele et al., 2000). Therefore, the immunostaining of the bead–neuron co-cultures with antibodies against the active zone marker Bassoon enabled the visualization of presynaptic differentiation on the surface of the NLGN1–coated beads (Figure 2A and Supplementary Figure 1A). In the control and GLA groups, wherein saline and GLA were administered to the mother, respectively, a marked accumulation of Bassoon signals was detected on the NLGN1–coated beads. However, the amount of the staining signals for Bassoon, i.e., the extent of presynaptic differentiation, was more modest in the GLA group than in the control group (*P* < 0.05; Figures 2A, B and Supplementary Figure 1B). The control beads coated with Fc exhibited no such pre-synapse-inducing activity in the control and GLA groups (Supplementary Figures 2A, B). There were no differences in background Bassoon signal intensity and *Bsn* mRNA expression levels between these two groups (Supplementary Figures 2C, D).

To examine the reproducibility and interlitter variations of the suppressive effect of maternal GLA exposure on the NLGN1-induced presynaptic differentiation, we compared results from five independent experiments (5 litters) at DIV13 alongside those from two independent experiments (2 litters) at DIV9. Figure 2C shows the ratio (%) of Bassoon signal intensity in the GLA group relative to that in the control group in each experiment. Although no difference in the NLGN1-induced presynaptic differentiation was observed between the GLA and control groups in one experiment (103%), the suppressive effect on the presynaptic differentiation exerted by the maternal exposure to GLA was reproduced in the remaining four experiments with an averaged Bassoon signal ratio of the GLA/control group of 48.6 ± 30.6% (mean ± SD) at DIV13. In addition, significant suppression of the NLGN1-induced presynaptic differentiation by the maternal GLA exposure was detected in two experiments of the synapse-formation assay performed at DIV9 (19 and 27%, respectively). These results suggest that maternal GLA exposure disturbs synapse formation and that the degree of the suppressive effect varies according to individual differences in the mother. We confirmed that the BW of pregnant mice was comparable between the control and GLA groups (Supplementary Figure 3A).

### 3.2 Presynaptic differentiation is unaltered via the subchronic administration of GLA to cultured cortical neurons

**FIGURE 3:**
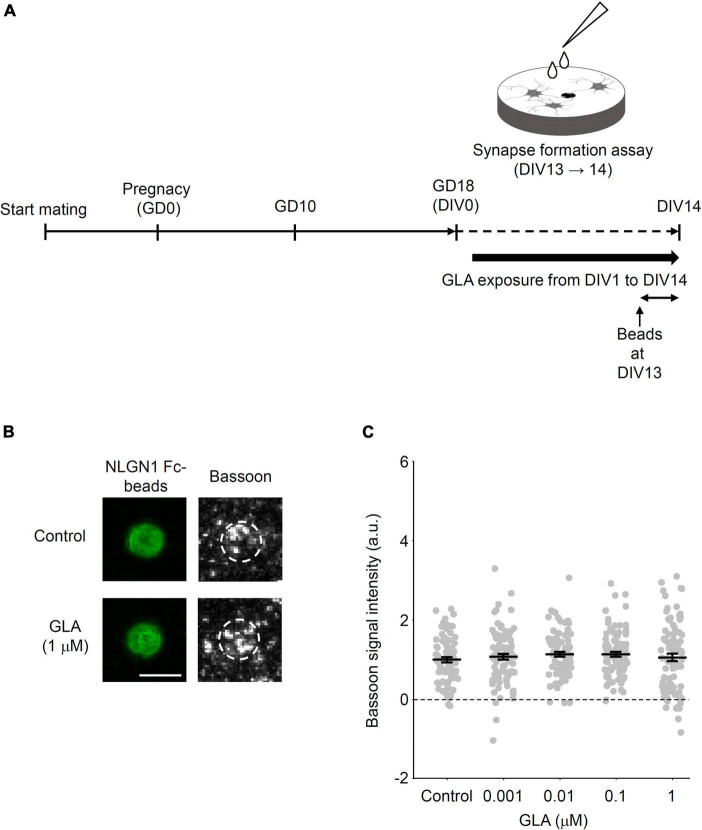
Little effects of subchronic direct GLA exposure on presynaptic differentiation. **(A)** Cultured cortical neurons from GD18 embryos without any treatments were prepared and exposed to vehicle (PBS) or GLA from DIV1 to DIV14. Synapse-formation assay was done at DIV13. **(B)** Representative images of Bassoon signals (gray) on NLGN1 Fc–coated beads (green) after subchronic direct GLA (1 μM) exposure or PBS (Control). White dashed circles outline beads. *Scale bar*, 5 μm **(C)** Intensity of Bassoon signals on the surface of NLGN1 Fc–coated beads [*n* = 80, 89, 89, 86, and 86 beads analyzed/3 cultures per litter/3 independent experiments for PBS (Control), 0.001, 0.01, 0.1, and 1 μM GLA groups, respectively]. Data are presented as dot plot of Bassoon signal intensity on NLGN1 Fc–coated beads in each experimental condition with mean ± SEM.

We showed that maternally administered GLA affected the presynaptic differentiation induced by NLGN1 in cultured cortical neurons obtained from embryos. Conversely, several groups reported the cellular outcomes via the bath application of GLA *in vitro*, including the *N*-methyl-D-aspartate receptor (NMDAR)-mediated activation of the neuronal network (Lantz et al., 2014) and impaired cell adhesion, cilia synthetization, and neuro-glial differentiation of neural stem cells (Feat-Vetel et al., 2018). Therefore, we next investigated whether direct GLA administration to cultured cortical neurons affects the NLGN1-induced presynaptic differentiation. Cultured cortical neurons were prepared from embryos in the same way as that shown in Figure 1A without any maternal treatment. Subsequently, the cultured neurons were chronically treated with a bath-applied GLA (0.001–1 μM) from DIV1 to DIV14 or with PBS alone in case of the control group (Figure 3A). Based on the previous report that the maximum plasma concentration of GLA 1 h after oral administration (2 mg/kg) to rats was 0.008 μg/g (∼0.040 μM) (Food Safety Commission of Japan, 2010, 2023; World Health Organization, 2021), the concentrations of GLA used in the present study for bath application were assumed to cover the range of the expected concentration in the embryonic brain in the maternal GLA exposure model (0.8 mg/kg) depicted in Figure 1A. The extent of NLGN1-induced presynaptic differentiation, as indicated by the accumulation of Bassoon signals on the beads, was comparable among the control vehicle–treated and GLA–treated groups at four different concentrations (Figures 3B, C). These results suggest that maternal GLA exposure decreases presynaptic differentiation in cultured cortical neurons obtained from the embryos through pathway(s) that are distinct from those underlying the direct action of GLA in neurons.

### 3.3 Maternal GLA exposure changes global gene expression in cultured cortical neurons during development

**FIGURE 4:**
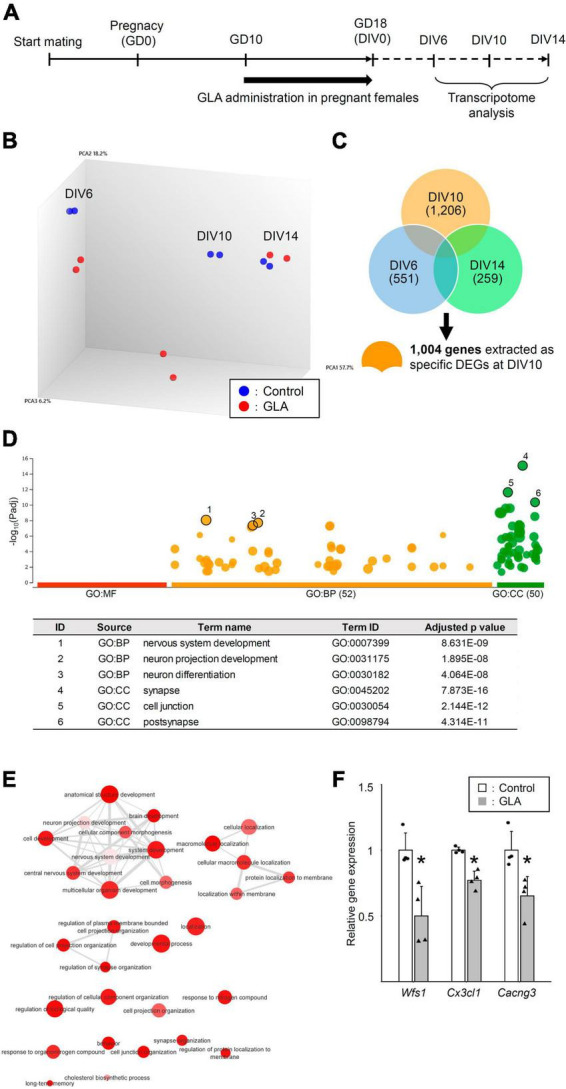
Global gene expression analysis of cultured neurons from maternally GLA exposed embryos. **(A)** Cultured cortical neurons were prepared along with Figure 1A. A transcriptome analysis was carried out with total RNA isolated from the neurons at DIV6, DIV10, and DIV14. **(B)** PCA on the comprehensive gene expression analysis data. PCA was performed using Transcriptome Analysis Console software. **(C)** Venn-diagrams of DEGs at DIV6, DIV10, and DIV14 with >1.3-fold change, and >10 average signal intensity of log value in the control group, without genes in sex chromosomes. Specific DEGs at DIV10 (1,004 genes) were extracted from the diagrams for GO enrichment analysis. **(D)** Downregulated DEGs at DIV10 relate to cell development. Manhattan plot illustrating GO analysis enriched in 374 downregulated DEGs at DIV10 (upper). The number in the source name in the *x*-axis labels shows how many significantly enriched terms were found in GO: MF (Molecular Function, red), GO: BP (Biological Process, orange) and GO: CC (Cellular Component, green). The *y*-axis shows the adjusted enrichment *p*-values and the circle size is proportional to the fold enrichment. Top 3 GO:BP (circle no. 1–3) and GO:CC (circle no. 4–6) annotations were tabulated (lower). **(E)** Interactive graph of BP enriched GO terms determined from downregulated DEGs. Circle colors indicate the *P*-value and its size indicates the frequency of the GO term in the underlying annotation database. Highly similar GO terms are connected by lines. **(F)** Confirmation of the microarray data by real-time quantitative PCR (RT-qPCR). The levels of expression of downregulated genes (*Wfs1*, *Cx3cl1*, and *Cacng3*) in the GLA and control groups were measured. Housekeeping gene, *Gapdh* was used for normalization and the data are presented as dot plot with mean ± SD (*n* = 4 each). *P < 0.05; two-tailed Student’s *t*-test.

Maternal GLA exposure–mediated interference in synapse formation in cultured neurons raised questions regarding the type of molecular changes that underlie this effect and the time point when the factors responsible for this effect become noticeable during neuronal development. Thus, we performed a transcriptome analysis of cultured cortical neurons from maternally control saline– or GLA–treated embryos at DIV6, DIV10, and DIV14 without the addition of NLGN1–coated beads (Figure 4A). A principal component analysis (PCA) revealed that cultured cortical neurons exhibited characteristic global gene expression profiles according to the developmental stage (Figure 4B). The maternal GLA–exposed group exhibited a similar profile to that of the control group at DIV6. In turn, the profile of the maternal GLA–exposed group was markedly different from that of the control group at DIV10 and became similar to that of the control group at DIV14. The extraction of the DEGs that exhibited a >1.3-fold expression change compared with the control group at each developmental stage led to the identification of 551, 1,206, and 259 DEGs at DIV6, DIV10, and DIV14, respectively (Figure 4C and Supplementary Table 1). A gene ontology (GO) enrichment analysis was performed using 1,004 specific DEGs at DIV10 (374 downregulated DEGs and 630 upregulated DEGs). The 374 downregulated DEGs were involved in 52 GO biological process terms (GO: BP) and 50 GO cellular component terms (GO: CC; Figure 4D and Supplementary Table 2). Figure 4E provides an interactive graph of 30 GO:BP with reduced redundancy obtained from the original GO terms in Figure 4D using the REVIGO tool (Supek et al., 2011). Maternal GLA exposure downregulated the genes involved in cellular pathways related to “nervous system development,” “synapse organization,” and higher brain functions (such as “long-term memory” and “behavior”). The expression levels of three genes related to these terms, *Wfs1*, *Cx3cl1*, and *Cacng3*, at DIV10 were confirmed via RT-qPCR (Figure 4F). Conversely, terms related to “metabolic process” among the 630 upregulated DEGs were obtained at DIV10 (Supplementary Figure 4). The transcriptome analysis indicated that genes related to nervous system development were downregulated in the maternal GLA–exposed group during neuronal development in our culture system. This suggests that maternal GLA exposure induces the dysregulation of neurodevelopment in the offspring.

### 3.4 Maternal GLA exposure impacts the early stage of cortical Pvalb (+) neuron development in the offspring

**FIGURE 5:**
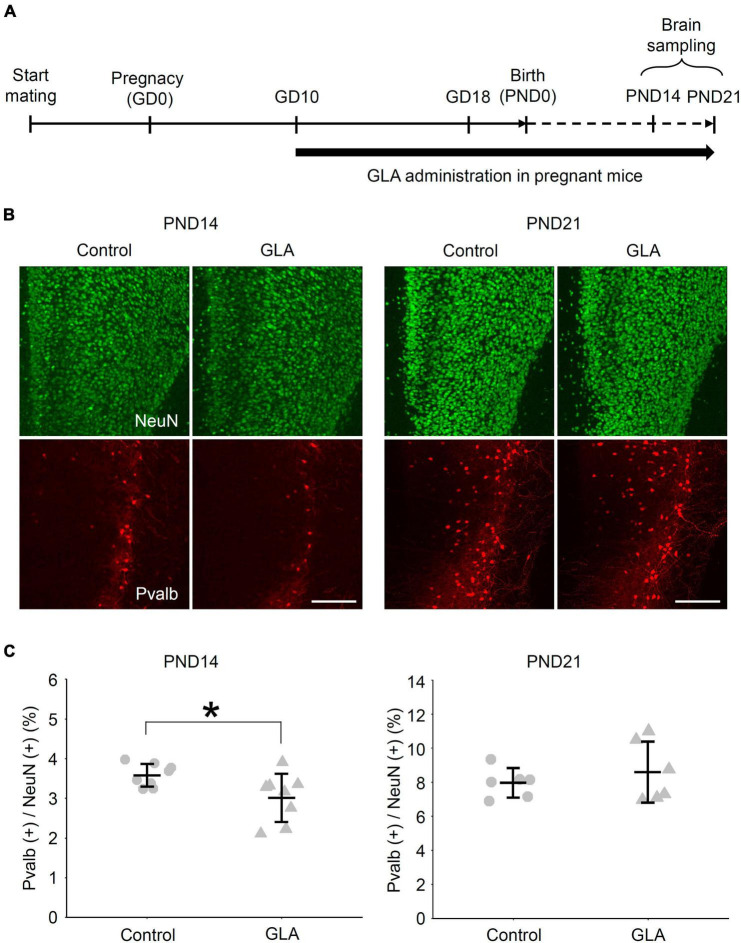
Effect of maternal GLA exposure on Pvalb (+) interneurons in the mPFC during postnatal development. **(A)** Experimental procedure of maternal GLA exposure. Pregnant mice were treated with GLA (0.8 mg/kg) from GD10 to PND21. Animals in the control group were treated with saline-vehicle. **(B)** Representative images of the distribution of NeuN- and Pvalb-immunoreactive cells in the mPFC of offspring from the control and GLA groups. Coronal sections of mouse mPFC at PND14 and PND21 from each group were subjected to immunostaining. *Scale bar*, 200 μm **(C)** Comparison of the densities of Pvalb (+) cells in the mPFC of offspring between maternally GLA exposed (GLA) and saline-vehicle (Control) groups (*n* = 8 each at PND14 and *n* = 6 each at PND21, respectively). Data are presented as dot plot with mean ± SD. *P < 0.05; two-tailed Student’s *t*-test.

The effect of maternal GLA exposure on the social behavior of the offspring has been documented including reduced ultrasonic vocalizations, social interaction, and social novelty preference (Laugeray et al., 2014; Dong et al., 2020). Nevertheless, information regarding the manner in which maternal GLA exposure affects the development of neuronal circuits in the brain remains scarce. In our transcriptome analysis, several genes related to Ca^2+^-binding protein *Pvalb* expression, such as *Camk1g* and *Gad1* (Cohen et al., 2016) were included among the 374 downregulated DEGs at DIV10 (Supplementary Table 1). The mPFC is of special importance for social cognition, and the decrease in Pvalb-immunoreactive [Pvalb (+)] interneurons in this cortical region has been implicated in neurodevelopmental disorders with social deficits, such as autism spectrum disorder (Filice et al., 2020). Lastly, we investigated the effect of maternal GLA exposure on Pvalb (+) interneuron development in the mPFC of the offspring (Figure 5A). The BW of offspring, as measured from PND0 to PND21, was comparable between the control and GLA groups (Supplementary Figure 3B). To determine whether Pvalb (+) interneuron development in the mPFC is affected by maternal GLA exposure, an immunofluorescence confocal microscopic analysis was performed. Figure 5B provides examples of Pvalb (+) neurons in the mPFC at PND14 and PND21. A slight but significant reduction in the density of Pvalb (+) neurons was detected in the GLA group (3.0 ± 0.6%) compared with that in the control group (3.6 ± 0.3%, *P* < 0.05) at PND14 (Figure 5C). However, no differences in the density of Pvalb (+) neurons between the GLA and control groups were observed at PND21, suggesting that the development of inhibitory interneuron circuits may be slightly delayed by maternal GLA exposure.

## 4 Discussion

The effects of pesticide exposure on synaptic differentiation and development have not been investigated in detail. Herein, we quantitatively evaluated the effect of GLA exposure on synapse formation in cultured cerebral cortical neurons using beads coated with a synaptic organizer, NLGN1. We observed that maternal GLA exposure reduced the accumulation of active zone protein Bassoon on the NLGN1–coated beads. In combination with a temporal gene expression analysis, we revealed the dysregulation of development in cultured neurons from fetuses that underwent maternal GLA exposure. Furthermore, the offspring of GLA–exposed mothers exhibited a lower density of Pvalb (+) interneurons in the mPFC at the postnatal developmental stage. Thus, these findings revealed the presence of a developmental synapse pathology following maternal exposure to GLA.

Herein, we demonstrated that the amount of newly formed synapses on NLGN1–coated beads decreased in cultured neurons as an effect of maternal GLA exposure on the CNS (Figure 2). However, the factors that decreased synapse formation remain unknown. It is well known that presynaptic differentiation induced by NLGN1 is mediated by presynaptic organizers, i.e., neurexins (Südhof, 2017).

The neurexin genes (*Nrxn1*, *Nrxn2*, and *Nrxn3*) are transcribed into two major isoforms (α-*Nrxn* and β-*Nrxn*) from distinct promoters, both of which exhibit numerous splice variants regulating synapse formation via binding with specific postsynaptic organizers based on the differences in the binding affinity of each variant. In particular, extensive studies have revealed that alternative splicing at splice site 4 (SS4) is regulated in a neuronal activity-dependent manner by RNA-binding proteins (i.e., Sam68, SLM1, and SLM2) (Iijima et al., 2011; Ding et al., 2017). Accordingly, dystroglycan located at the postsynaptic membrane binds to specific laminin-neurexin-sex hormone-binding globulin domains of several NRXN3 splice variants to control the inhibitory synapse function (Trotter et al., 2023). Herein, the genes that encode SLM2 and dystroglycan (*Khdrbs3* and *Dag1*, respectively) were included in the DEGs detected at DIV10. Furthermore, “pre-synapse” with 30 genes (e.g., *Napb*, *Rab3a*, and *Snap25*) was identified as a GO:CC in the GO analysis among the downregulated DEGs (Supplementary Table 2). Therefore, these maternal GLA exposure–mediated changes in the expression of genes encoding for the neurexin-related proteins and key regulators of the presynaptic transmitter release machinery may affect selective *trans-*synaptic interactions and excitatory/inhibitory synaptic balances, as measured using synapse-formation assays with NLGN1-beads.

Based on these *in vitro* results, we also detected a decrease in the density of Pvalb (+) interneurons in the mPFC at PND14 in the offspring that underwent maternal GLA exposure, which was subsequently indistinguishable from that of the saline-control group at PND21 (Figure 5). Pvalb interneurons were actually presented in the cortex long before they begin to express detectable level of Pvalb (Filice et al., 2020). Considering that genes related to Pvalb expression (*Camk1g* and *Gad1*) were downregulated at DIV10 (Supplementary Table 2), our results might reflect a delay in *Pvalb* expression rather than a loss of Pvalb interneurons. Laugeray et al. (2014) reported perinatal GLA exposure contributes to abnormal sociability, which is a characteristic behavioral change observed in mouse models of autism. The lower density of Pvalb (+) interneurons observed in the offspring with maternal GLA exposure could be considered as an intermediate phenotype leading to such behavioral outcomes. A transient change in the density of Pvalb (+) neurons and inhibitory synapses during the developmental stage, which becomes undetectable in adults, can result in a long-lasting behavioral abnormality (Magno et al., 2021). Overall, our results suggest that maternal GLA exposure caused dysregulation in brain development, which may not be discernable in the adult stage. Interestingly, significant GO terms related to “developmental delay” were obtained from the Human Phenotype Ontology database (Köhler et al., 2014), with 374 downregulated DEGs detected at DIV10 (Supplementary Table 3).

Furthermore, this suppressive effect on presynaptic differentiation was not observed when a similar dose of GLA to that received through their mother was applied directly to cultured cortical neurons from the fetuses (Figure 3). Regarding developmental toxicity during pregnancy, three main pathways via which pesticides affect fetal development are considered (Rogers and Kavlock, 2001). First, pesticides can pass through the placenta and act directly on the fetus. Second, maternal pesticide exposure results in placental dysfunction (reduced size or blood flow and altered transport or metabolism). Third, developmental toxicity can be attributed to maternal effects (anemia, malnutrition, electrolyte imbalance, decreased uterine blood flow, changes in endocrine function and gut microbiota, etc.), which alter the maternal susceptibility to pesticides. In general, a combination of these pathways can trigger various fetal developmental abnormalities. In fact, the second and third pathways are more likely to contribute to the developmental synapse pathology observed in the current study. Herein, maternal GLA exposure during gestation exhibited no effect on the weight of fetuses and their mothers (Supplementary Figure 3). These results indicate that the dysregulation of neurodevelopment is not caused by the deterioration of the parenting environment. Previous studies have reported that the effects of GLA exposure include the activation of NMDAR (Lantz et al., 2014), inhibition of GS (Menga et al., 2020), increase in astrocytes density (Meme et al., 2009), induction of inflammation depending on the IL-1 receptor (Maillet et al., 2016), and change in the intestinal flora (Dong et al., 2020). By analyzing the maternal and placental changes caused by maternal GLA exposure and by identifying the main factors and/or pathways involved in this phenomenon by verifying their relationship with the developmental synapse pathology, we would be able to clarify the process underlying the developmental synapse pathology and identify new therapeutic targets. Recent studies regarding the details of the action mechanism of GLA in plants may also provide hints that can be used for further research (Takano and Dayan, 2020). Exposure to other pesticides also reportedly increases the risk of neurodevelopmental disorders (von Ehrenstein et al., 2019). Furthermore, maternal abnormalities, such as pre-eclampsia and maternal immune activation triggered by infection, are thought to increase this risk (Walker et al., 2015; Xie et al., 2020; Hall et al., 2023). Therefore, it would be reasonable to assume that a common pathway involving inflammatory cytokines (IL-1β, IL-6, and TNF-α) underlies these maternal effects on fetal neurodevelopment, including the developmental synapse pathology in part.

Based on the oral GLA administration model as mentioned above (Food Safety Commission of Japan, 2010, 2023; World Health Organization, 2021), we estimated the blood concentration in our maternal GLA exposure model (0.8 mg/kg BW) to be 0.016 μM. Although the concentration of GLA transferred into the blood likely depends on administration routes, such as oral and intranasal, as well as on the species, including rat and mouse, the estimated GLA concentration in the fetal brain in our model is lower than that causing neurotoxicity at the range of 10–1000 μM reported by Lantz et al. (2014) and Feat-Vetel et al. (2018). Some GLA metabolites with the ranges of 100–300 μM for N-acetyl glufosinate and 3–100 μM for 4-methylphosphinico-2-oxobutanoic acid are known to lead to similar neurotoxicity (Lantz et al., 2014 and Feat-Vetel et al., 2018). Therefore, further detailed analysis of the amount of GLA and its metabolites transferred into fetus brain is required to better understand the developmental synapses pathology by maternal GLA exposure.

## Data availability statement

The original contributions presented in the study are included in the article/Supplementary material, further inquiries can be directed to the corresponding author.

## Ethics statement

The animal study was approved by the Animal Experiment Committee of the University of Toyama. This study was conducted in accordance with the local legislation and institutional requirements.

## Author contributions

HI: Conceptualization, Data curation, Formal analysis, Funding acquisition, Investigation, Methodology, Resources, Writing – original draft. MD: Formal analysis, Writing – review and editing. AI: Data curation, Formal analysis, Writing – review and editing. RO: Data curation, Formal analysis, Writing – review and editing. MF: Methodology, Writing – review and editing. TO: Methodology, Software, Writing – review and editing. TT: Methodology, Software, Writing – review and editing. HM: Conceptualization, Resources, Supervision, Visualization, Writing – review and editing. TY: Conceptualization, Data curation, Formal analysis, Funding acquisition, Investigation, Methodology, Project administration, Validation, Visualization, Writing – original draft.

## Supplementary material

Supplementary Figure 1 Comparison of Bassoon signals on NLGN1-Fc beads between Control and GLA groups. **(A)** Representative images of Bassoon signal intensity (gray) on NLGN1 Fc–coated beads (white dashed circle) in Control and GLA groups. Images are sorted in ascending order by Bassoon accumulation on the beads in each experimental condition. *Scale bar*, 5 μm **(B)** Change in cumulative distribution of Bassoon signals on NLGN1 Fc–coated beads by maternal GLA exposure (control; dashed line and GLA groups; solid line).

Supplementary Figure 2 No presynaptic differentiation on Fc beads. **(A)** Representative images of Bassoon signals (gray) on control Fc–coated beads (green) co-cultured with neurons prepared with the same procedure shown in Figure 1. White dashed circles outline beads. *Scale bar*, 5 μm **(B)** Intensity of Bassoon signals on Fc–coated beads (*n* = 56 and 78 beads analyzed/3 cultures per litter/3 individual experiments for Control and GLA group, respectively). Data are presented as dot plot of Bassoon signal intensity on Fc–coated beads in each experimental condition with mean ± SEM. **(C)** No difference in background Bassoon signal intensity without NLGN1 Fc–coated beads between control and GLA groups. Data are presented as dot plot with mean ± SD (*n* = 5 independent experiments). **(D)** Little effect by maternal GLA exposure on Bassoon (*Bsn*) mRNA expression. *Bsn* expression levels of cultured neurons from maternally GLA– and mock–treated embryos were estimated by real-time PCR. Data are presented as dot plot with mean ± SD (*n* = 4 independent experiments each).

Supplementary Figure 3 No effect of GLA exposure on the body weight of mothers and offspring during pregnancy and postnatal developmental stage. **(A)** Changes in body weight of pregnant mice by GLA administration at GD10, GD12, GD14, GD15, and GD17. Body weight of the pregnant mice was not influenced by GLA exposure (*n* = 10 and 7 for Control and GLA group, respectively). **(B)** Changes in body weight of the offspring mice at PND0, PND7, PND14, and PND21. There was no difference in body weight between maternally GLA-exposed (GLA) and saline-vehicle (Control) groups (*n* = 12 and 16 for Control and GLA group, respectively). Data are presented as mean ± SD.

Supplementary Figure 4 Upregulated DEGs at DIV10 relate to cell metabolic process. **(A)** Manhattan plot illustrating GO analysis enriched in 630 upregulated DEGs at DIV10 (upper). The number of enriched GO terms were shown with the source name (MF, BP, and CC) in the *x*-axis labels. Circle size is proportional to the fold enrichment and color is proportional to significance. Top 3 GO:BP (circle no. 1–3) and GO:CC (circle no. 4–6) annotations were tabulated (lower). **(B)** Interactive graph of BP enriched GO terms determined from upregulated genes. Circle colors indicate the *P*-value and its size indicates the frequency of the GO term in the underlying annotation database. Highly similar GO terms are connected by lines.
