# Supplementary figures and images for: Developmental synapse pathology triggered by maternal exposure to the herbicide glufosinate ammonium
Source: Front Mol Neurosci. 2023 Nov 30;16:1298238. doi: 10.3389/fnmol.2023.1298238 (PMC10720911; doi:10.3389/fnmol.2023.1298238)

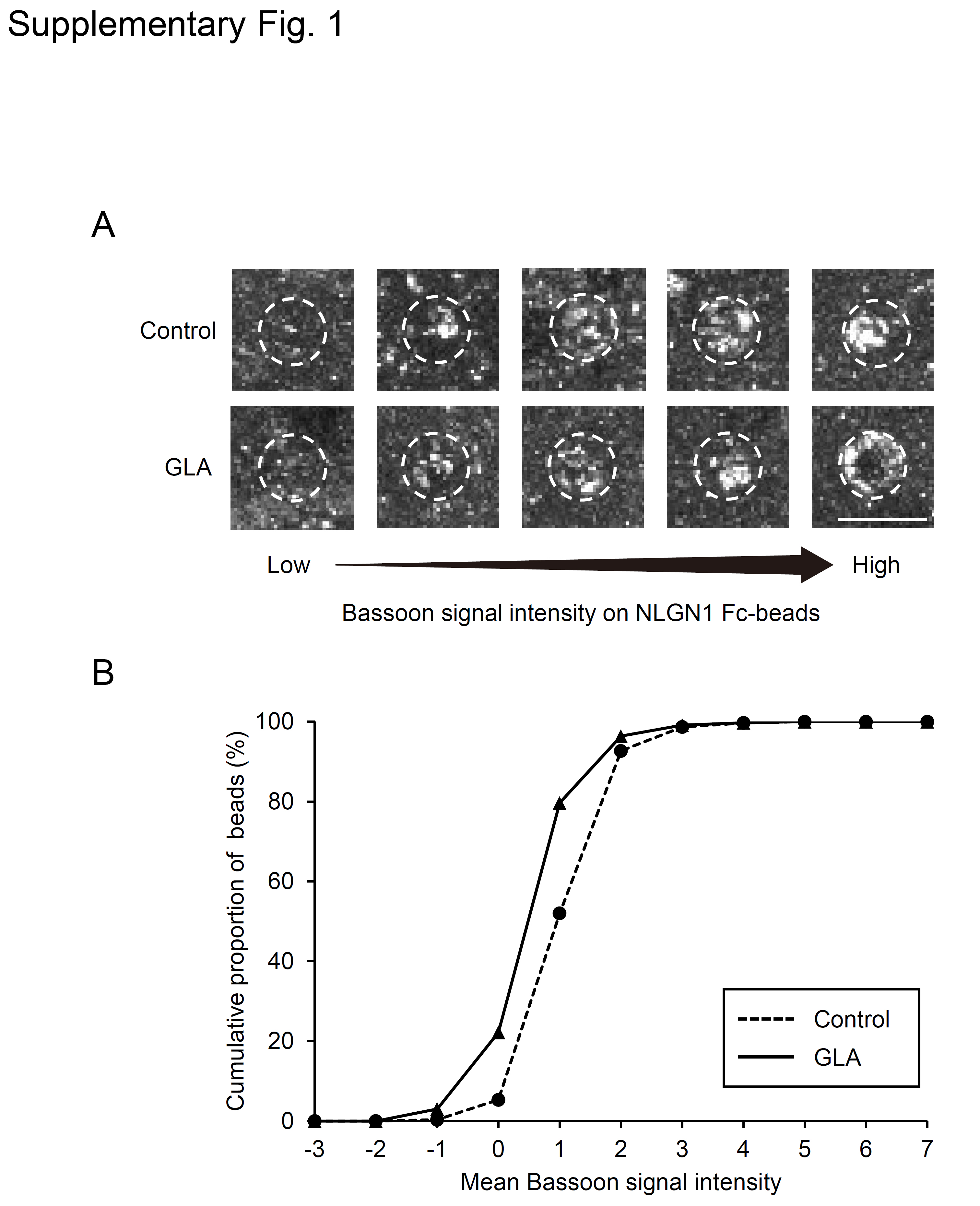

Supplement: Supplementary Figure 1 — Comparison of Bassoon signals on NLGN1-Fc beads between Control and GLA groups. (A) Representative images of Bassoon signal intensity (gray) on NLGN1 Fc–coated beads (white dashed circle) in Control and GLA groups. Images are sorted in ascending order by Bassoon accumulation on the beads in each experimental condition. Scale bar, 5 μm (B) Change in cumulative distribution of Bassoon signals on NLGN1 Fc–coated beads by maternal GLA exposure (control; dashed line and GLA groups; solid line). [file Image_1.jpg]

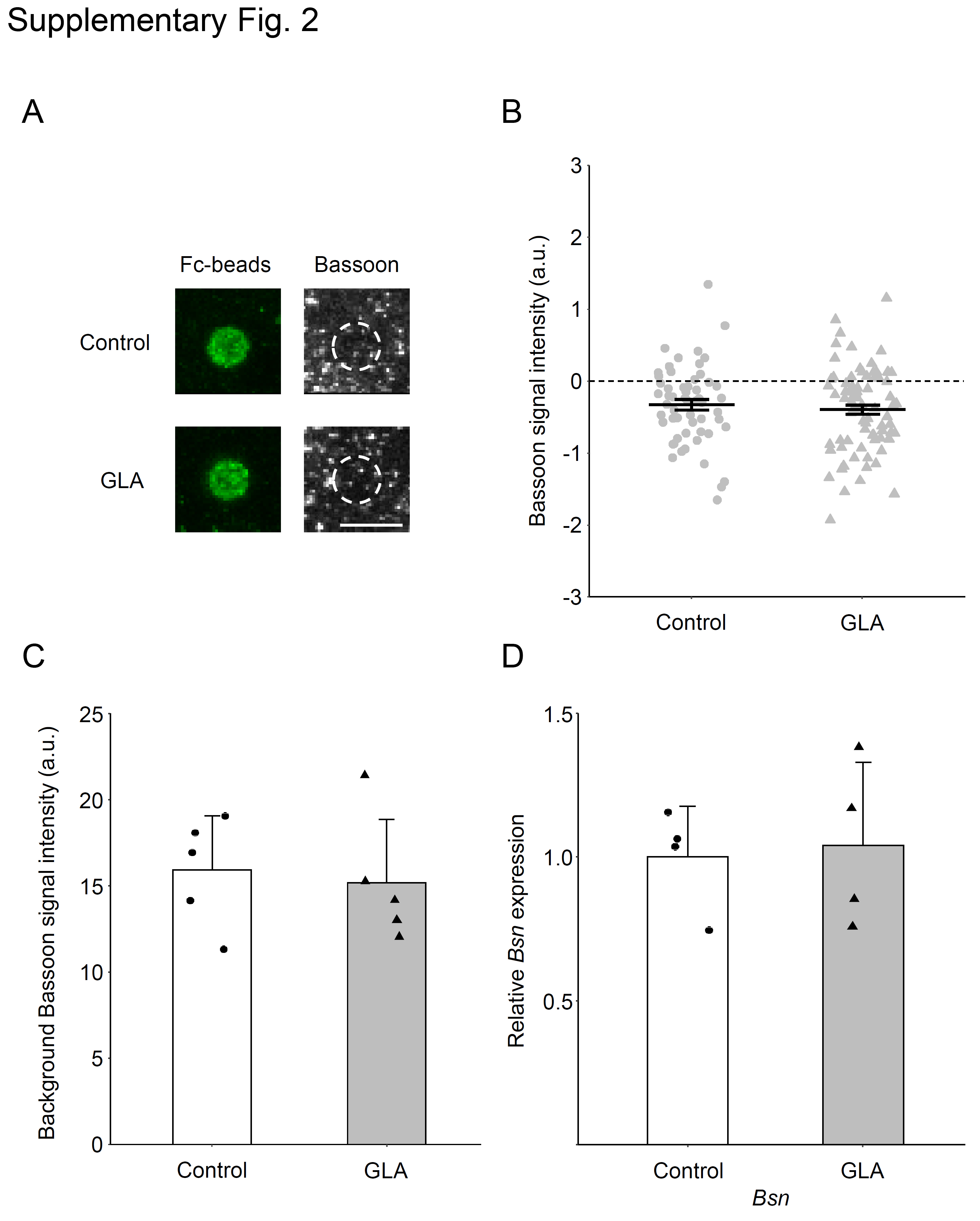

Supplement: Supplementary Figure 2 — No presynaptic differentiation on Fc beads. (A) Representative images of Bassoon signals (gray) on control Fc–coated beads (green) co-cultured with neurons prepared with the same procedure shown in Figure 1. White dashed circles outline beads. Scale bar, 5 μm (B) Intensity of Bassoon signals on Fc–coated beads (n = 56 and 78 beads analyzed/3 cultures per litter/3 individual experiments for Control and GLA group, respectively). Data are presented as dot plot of Bassoon signal intensity on Fc–coated beads in each experimental condition with mean ± SEM. (C) No difference in background Bassoon signal intensity without NLGN1 Fc–coated beads between control and GLA groups. Data are presented as dot plot with mean ± SD (n = 5 independent experiments). (D) Little effect by maternal GLA exposure on Bassoon (Bsn) mRNA expression. Bsn expression levels of cultured neurons from maternally GLA– and mock–treated embryos were estimated by real-time PCR. Data are presented as dot plot with mean ± SD (n = 4 independent experiments each). [file Image_2.jpg]

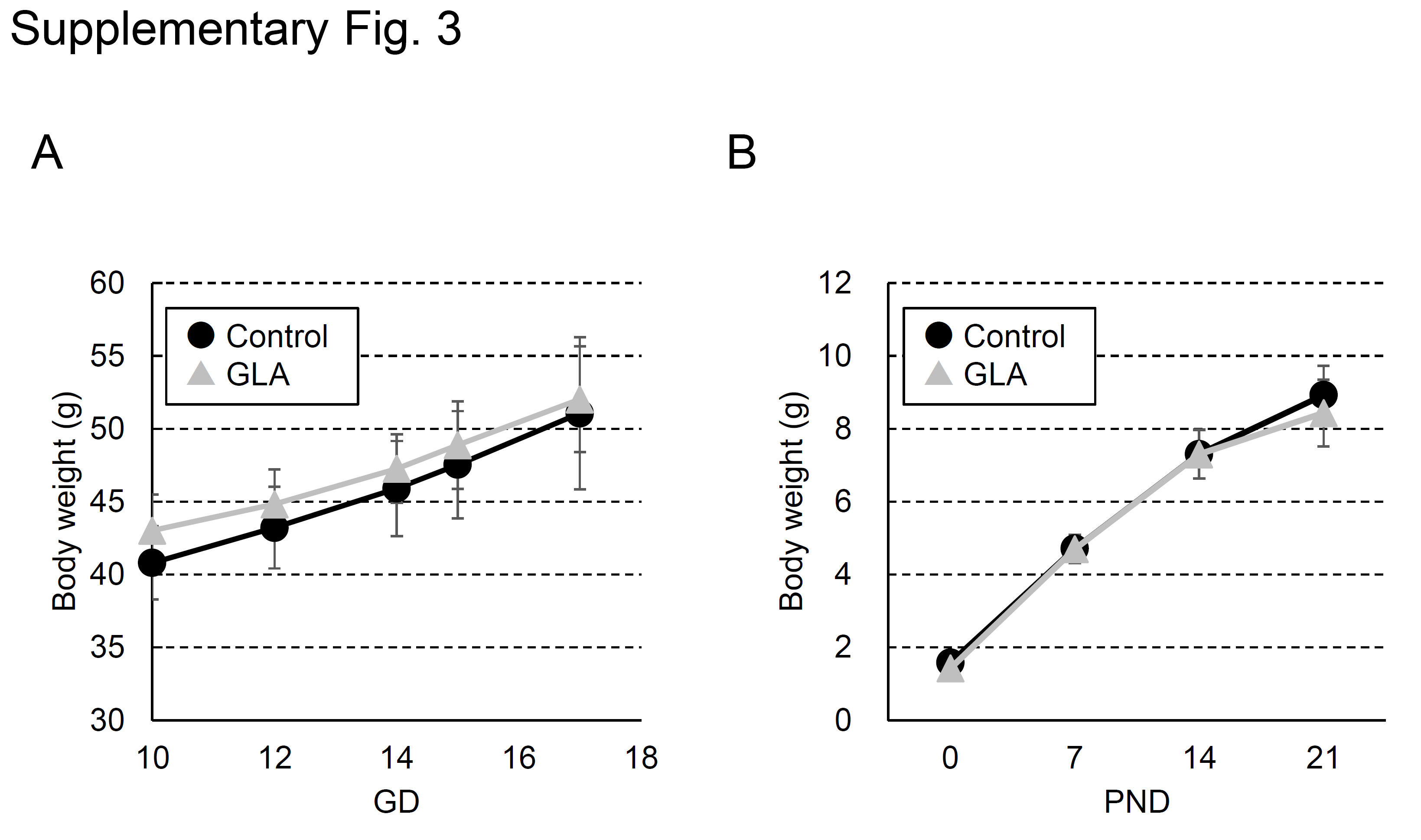

Supplement: Supplementary Figure 3 — No effect of GLA exposure on the body weight of mothers and offspring during pregnancy and postnatal developmental stage. (A) Changes in body weight of pregnant mice by GLA administration at GD10, GD12, GD14, GD15, and GD17. Body weight of the pregnant mice was not influenced by GLA exposure (n = 10 and 7 for Control and GLA group, respectively). (B) Changes in body weight of the offspring mice at PND0, PND7, PND14, and PND21. There was no difference in body weight between maternally GLA-exposed (GLA) and saline-vehicle (Control) groups (n = 12 and 16 for Control and GLA group, respectively). Data are presented as mean ± SD. [file Image_3.jpg]

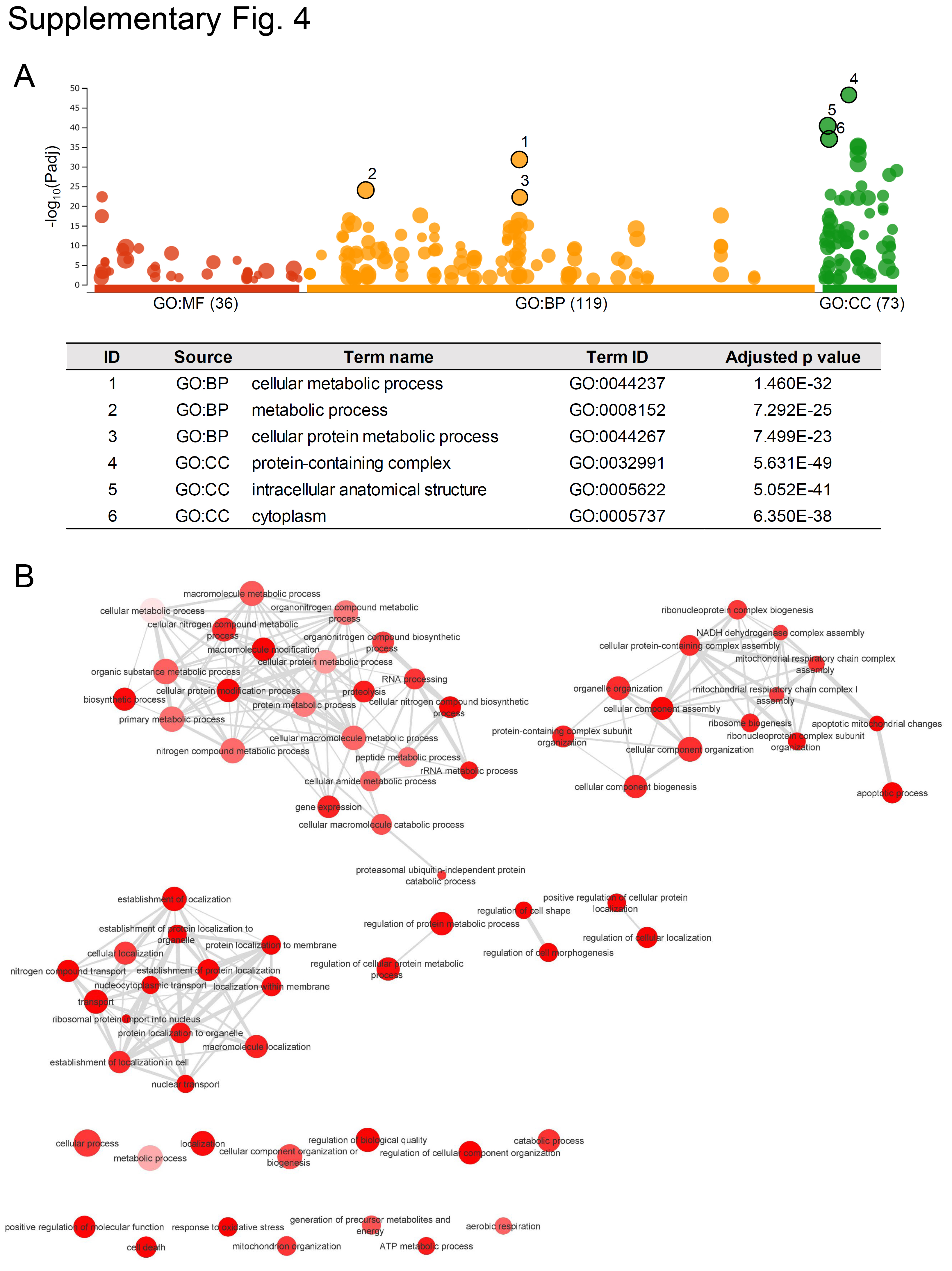

Supplement: Supplementary Figure 4 — Upregulated DEGs at DIV10 relate to cell metabolic process. (A) Manhattan plot illustrating GO analysis enriched in 630 upregulated DEGs at DIV10 (upper). The number of enriched GO terms were shown with the source name (MF, BP, and CC) in the x-axis labels. Circle size is proportional to the fold enrichment and color is proportional to significance. Top 3 GO:BP (circle no. 1–3) and GO:CC (circle no. 4–6) annotations were tabulated (lower). (B) Interactive graph of BP enriched GO terms determined from upregulated genes. Circle colors indicate the P-value and its size indicates the frequency of the GO term in the underlying annotation database. Highly similar GO terms are connected by lines. [file Image_4.jpg]
